# (*E*)-2-{3-[4-(Diphenyl­amino)styr­yl]-5,5-dimethyl­cyclo­hex-2-enyl­idene}­malono­nitrile

**DOI:** 10.1107/S1600536809014378

**Published:** 2009-04-25

**Authors:** Hai-Dong Ju, Xu-Tang Tao, Shi-Qing Xu, Wen-Tao Yu

**Affiliations:** a College of Materials Science and Engineering, China Jiliang University, Hangzhou 310018, People’s Republic of China; b State Key Laboratory of Crystalline Materials, Shandong University, Jinan 250100, People’s Republic of China

## Abstract

In the title compound, C_31_H_27_N_3_, the cyclo­hexene ring has an envelope configuration. In the crystal structure, there is an 34 Å^3^ void around the inversion center, but the low electron density (0.13 e Å^−3^) in the difference Fourier map suggests no solvent mol­ecule occupying this void. No hydrogen bonding is found in the crystal structure.

## Related literature

For background to organic compounds with light emitting properties, see: Tang *et al.* (1998 ▶); Li *et al.* (2003 ▶); Hye *et al.* (2004 ▶). For the synthesis, see: Lemke (1974 ▶); Tao & Miyata (2001 ▶). For related crystal structures, see: Kia *et al.* (2009 ▶); Ju *et al.* (2006 ▶).
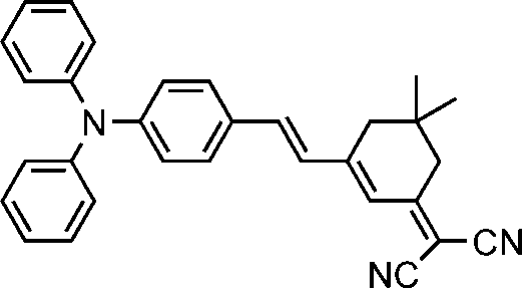


## Experimental

### 

#### Crystal data

C_31_H_27_N_3_
*M* *_r_* = 441.56
Monoclinic, 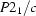

*a* = 13.239 (5) Å
*b* = 16.757 (8) Å
*c* = 11.886 (3) Å
β = 104.073 (5)°
*V* = 2557.7 (17) Å^3^
*Z* = 4
Mo *K*α radiation
μ = 0.07 mm^−1^
*T* = 293 K
0.48 × 0.19 × 0.16 mm

#### Data collection

Bruker SMART area-detector diffractometer
Absorption correction: none
20681 measured reflections
5879 independent reflections
3554 reflections with *I* > 2σ(*I*)
*R*_int_ = 0.027

#### Refinement

*R*[*F* ^2^ > 2σ(*F* ^2^)] = 0.046
*wR*(*F* ^2^) = 0.167
*S* = 0.92
5879 reflections
309 parameters
H-atom parameters constrained
Δρ_max_ = 0.13 e Å^−3^
Δρ_min_ = −0.18 e Å^−3^

### 

Data collection: *SMART* (Bruker, 2002 ▶); cell refinement: *SAINT* (Bruker, 2002 ▶); data reduction: *SAINT*; program(s) used to solve structure: *SHELXS97* (Sheldrick, 2008 ▶); program(s) used to refine structure: *SHELXL97* (Sheldrick, 2008 ▶); molecular graphics: *ORTEP-3 for Windows* (Farrugia, 1997 ▶); software used to prepare material for publication: *WinGX* (Farrugia, 1999 ▶).

## Supplementary Material

Crystal structure: contains datablocks global, I. DOI: 10.1107/S1600536809014378/xu2505sup1.cif

Structure factors: contains datablocks I. DOI: 10.1107/S1600536809014378/xu2505Isup2.hkl

Additional supplementary materials:  crystallographic information; 3D view; checkCIF report

## Figures and Tables

**Fig. 1.**
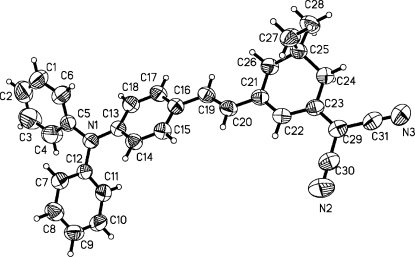
The molecular structure of the molecular structure of (I) showing the atom labels. Displacement ellipsoids are shown at the 50%.

**Fig. 2.**
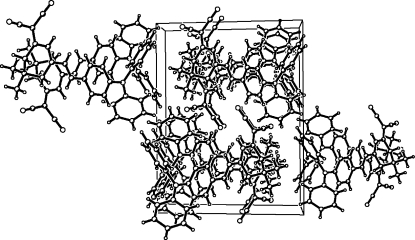
The packing of (I), viewed down the b axis. H atoms not involved in hydrogen bonding have been omitted.

## supplementary crystallographic information

### Comment

The organic compounds with donor-π-acceptor (D-π-A) structure have special light-emitting properties, and show potential application in organic light-emitting diodes (Tang *et al.*, 1998; Hye *et al.*, 2004). However, these molecules easily aggregate, which usually reduces their fluorescence intensity (Li *et al.*, 2003). Therefore, it is important to study their intermolecular interaction in the solid state. Recently we synthesized the title compound and studied its crystal structure.

The molecular structure is shown in Fig. 1. Three benzenes of triphenyl amine group show a three-bladed propeller configuration due to repulsion force. The bond lengths of C13—N1 are shorter than the single C—N distance (1.47–1.50 Å) and longer than double C=N bond distance (1.34–1.38 Å), which is due to the conjugation of *p*-π in triphenyl amine group. Because of long conjugation length, all atoms are roughly coplanar (Kia *et al.*, 2009). However, the cyclohexene group shows an envelope configuration due to its ring tension, which atoms are partly out of the plane (Ju *et al.*, 2006). The triphenyl amine and cyclohexene groups could hold back farther gather of these molecules in the solid state, which is due to their non-coplanar conjugation. No hydrogen bonding is found in the crystal structure (Fig. 2).

### Experimental

Hexahydropyridine (1 ml) and acetic acid (2 ml) were respectively added dropwise to a stirred benzene (100 ml) solution with 4-diphenylamino-benzaldehyde (1.1 g, 4 mmol) and 2-(3,5,5-Trimethylcyclohex-2-enylidene)-malononitrile (0.92 g, 5 mmol). The mixture was stirred at room temperature for 1 h, then separated water at refluxing temperature for another 5 h. Cooled to room temperature, the title compound was gotten (Lemke, 1974; Tao & Miyata, 2001). Single crystal suitable for X-ray diffraction analysis were obtained by slow evaporation of its ethanol saturated solution at room temperature.

### Refinement

All H atoms were positioned geometrically, and allowed to ride on their parent atom with C—H = 0.93 (aromatic), 0.96 (methyl) and 0.97 Å (methylene). *U*_iso_(H) = 1.5*U*_eq_(C) for methyl group and 1.2*U*_eq_(C) for others.

### Crystal data

**Table:**

C_31_H_27_N_3_	*F*(000) = 936
*M**_r_* = 441.56	*D*_x_ = 1.147 Mg m^−^^3^
Monoclinic, *P*2_1_/*c*	Mo *K*α radiation, λ = 0.71069 Å
Hall symbol: -P 2ybc	Cell parameters from 962 reflections
*a* = 13.239 (5) Å	θ = 2.4–20.0°
*b* = 16.757 (8) Å	µ = 0.07 mm^−^^1^
*c* = 11.886 (3) Å	*T* = 293 K
β = 104.073 (5)°	Block, red
*V* = 2557.7 (17) Å^3^	0.48 × 0.19 × 0.16 mm
*Z* = 4	

### Data collection

**Table:**

Bruker SMART area-detector diffractometer	3554 reflections with *I* > 2σ(*I*)
Radiation source: fine-focus sealed tube	*R*_int_ = 0.027
graphite	θ_max_ = 27.5°, θ_min_ = 1.6°
φ and ω scans	*h* = −17→17
20681 measured reflections	*k* = −21→20
5879 independent reflections	*l* = −15→15

### Refinement

**Table:**

Refinement on *F*^2^	Primary atom site location: structure-invariant direct methods
Least-squares matrix: full	Secondary atom site location: difference Fourier map
*R*[*F*^2^ > 2σ(*F*^2^)] = 0.046	Hydrogen site location: inferred from neighbouring sites
*wR*(*F*^2^) = 0.167	H-atom parameters constrained
*S* = 0.92	*w* = 1/[σ^2^(*F*_o_^2^) + (0.1*P*)^2^ + 0.187*P*] where *P* = (*F*_o_^2^ + 2*F*_c_^2^)/3
5879 reflections	(Δ/σ)_max_ < 0.001
309 parameters	Δρ_max_ = 0.13 e Å^−^^3^
0 restraints	Δρ_min_ = −0.18 e Å^−^^3^

### Special details

**Table:**

Geometry. All e.s.d.'s (except the e.s.d. in the dihedral angle between two l.s. planes) are estimated using the full covariance matrix. The cell e.s.d.'s are taken into account individually in the estimation of e.s.d.'s in distances, angles and torsion angles; correlations between e.s.d.'s in cell parameters are only used when they are defined by crystal symmetry. An approximate (isotropic) treatment of cell e.s.d.'s is used for estimating e.s.d.'s involving l.s. planes.
Refinement. Refinement of F^2^ against ALL reflections. The weighted R-factor wR and goodness of fit S are based on F^2^, conventional R-factors R are based on F, with F set to zero for negative F^2^. The threshold expression of F^2^ > 2sigma (F^2^) is used only for calculating R-factors (gt) etc. and is not relevant to the choice of reflections for refinement. R-factors based on F^2^ are statistically about twice as large as those based on F, and R- factors based on ALL data will be even larger.

### Fractional atomic coordinates and isotropic or equivalent isotropic displacement parameters (Å^2^)

**Table:**

	*x*	*y*	*z*	*U*_iso_*/*U*_eq_	
N1	0.81570 (11)	0.18274 (8)	0.93407 (11)	0.0627 (4)	
N2	0.43530 (17)	0.55197 (11)	0.20566 (18)	0.1061 (6)	
N3	0.21605 (14)	0.45416 (10)	−0.09927 (15)	0.0845 (5)	
C1	0.7581 (2)	0.01018 (13)	1.1028 (2)	0.0974 (7)	
H1	0.7009	−0.0186	1.1131	0.117*	
C2	0.8549 (3)	−0.01085 (14)	1.1632 (2)	0.1187 (10)	
H2	0.8641	−0.0534	1.2148	0.142*	
C3	0.9377 (2)	0.03051 (15)	1.1477 (2)	0.1226 (10)	
H3	1.0043	0.0163	1.1891	0.147*	
C4	0.92488 (17)	0.09351 (13)	1.07133 (19)	0.0943 (7)	
H4	0.9830	0.1213	1.0615	0.113*	
C5	0.82738 (14)	0.11594 (10)	1.00942 (13)	0.0585 (4)	
C6	0.74278 (16)	0.07411 (11)	1.02576 (16)	0.0741 (5)	
H6	0.6758	0.0884	0.9856	0.089*	
C7	0.86935 (13)	0.28927 (10)	1.07559 (14)	0.0600 (4)	
H7	0.8285	0.2669	1.1209	0.072*	
C8	0.92347 (14)	0.35847 (11)	1.11063 (15)	0.0692 (5)	
H8	0.9191	0.3828	1.1796	0.083*	
C9	0.98393 (15)	0.39192 (11)	1.04424 (17)	0.0719 (5)	
H9	1.0205	0.4388	1.0682	0.086*	
C10	0.99010 (14)	0.35617 (12)	0.94293 (16)	0.0742 (5)	
H10	1.0304	0.3791	0.8975	0.089*	
C11	0.93693 (14)	0.28622 (11)	0.90749 (15)	0.0670 (5)	
H11	0.9425	0.2616	0.8392	0.080*	
C12	0.87536 (12)	0.25291 (9)	0.97371 (13)	0.0531 (4)	
C13	0.73979 (12)	0.18623 (9)	0.82857 (12)	0.0521 (4)	
C14	0.69523 (13)	0.25939 (9)	0.78731 (13)	0.0543 (4)	
H14	0.7134	0.3051	0.8321	0.065*	
C15	0.62532 (12)	0.26470 (9)	0.68204 (14)	0.0532 (4)	
H15	0.5975	0.3143	0.6563	0.064*	
C16	0.59430 (11)	0.19754 (9)	0.61181 (13)	0.0504 (4)	
C17	0.63661 (12)	0.12448 (10)	0.65608 (13)	0.0571 (4)	
H17	0.6158	0.0784	0.6131	0.069*	
C18	0.70818 (13)	0.11848 (10)	0.76140 (14)	0.0578 (4)	
H18	0.7355	0.0689	0.7878	0.069*	
C19	0.52268 (12)	0.20214 (10)	0.49796 (13)	0.0533 (4)	
H19	0.4978	0.1541	0.4626	0.064*	
C20	0.48912 (12)	0.26930 (10)	0.43923 (14)	0.0552 (4)	
H20	0.5141	0.3171	0.4753	0.066*	
C21	0.41880 (11)	0.27527 (9)	0.32670 (13)	0.0501 (4)	
C22	0.40255 (12)	0.34749 (9)	0.27357 (13)	0.0549 (4)	
H22	0.4357	0.3918	0.3131	0.066*	
C23	0.33747 (12)	0.35870 (9)	0.16069 (13)	0.0512 (4)	
C24	0.28127 (13)	0.28808 (9)	0.09855 (13)	0.0561 (4)	
H24A	0.3210	0.2671	0.0465	0.067*	
H24B	0.2144	0.3056	0.0515	0.067*	
C25	0.26297 (12)	0.22075 (9)	0.17857 (13)	0.0520 (4)	
C26	0.36565 (12)	0.20259 (9)	0.26656 (14)	0.0545 (4)	
H26A	0.3521	0.1660	0.3243	0.065*	
H26B	0.4123	0.1761	0.2269	0.065*	
C27	0.17984 (13)	0.24505 (11)	0.24035 (16)	0.0686 (5)	
H27A	0.1163	0.2569	0.1840	0.103*	
H27B	0.2026	0.2915	0.2869	0.103*	
H27C	0.1681	0.2021	0.2891	0.103*	
C28	0.22779 (15)	0.14665 (11)	0.10523 (16)	0.0787 (6)	
H28A	0.2813	0.1307	0.0679	0.118*	
H28B	0.1651	0.1584	0.0475	0.118*	
H28C	0.2148	0.1042	0.1541	0.118*	
C29	0.32897 (13)	0.43195 (9)	0.10715 (14)	0.0590 (4)	
C30	0.38730 (16)	0.49914 (11)	0.16151 (17)	0.0730 (5)	
C31	0.26565 (15)	0.44413 (10)	−0.00723 (17)	0.0649 (5)	

### Atomic displacement parameters (Å^2^)

**Table:**

	*U*^11^	*U*^22^	*U*^33^	*U*^12^	*U*^13^	*U*^23^
N1	0.0691 (9)	0.0547 (8)	0.0565 (8)	−0.0110 (7)	−0.0001 (7)	0.0077 (6)
N2	0.1275 (16)	0.0607 (11)	0.1231 (15)	−0.0240 (11)	0.0169 (12)	−0.0041 (10)
N3	0.1009 (13)	0.0683 (11)	0.0790 (11)	−0.0048 (9)	0.0118 (9)	0.0167 (8)
C1	0.141 (2)	0.0771 (15)	0.0818 (15)	−0.0301 (15)	0.0420 (15)	0.0037 (11)
C2	0.190 (3)	0.0671 (15)	0.0774 (15)	−0.0170 (18)	−0.0100 (18)	0.0142 (11)
C3	0.133 (2)	0.0778 (16)	0.122 (2)	−0.0014 (16)	−0.0367 (17)	0.0290 (15)
C4	0.0822 (14)	0.0771 (14)	0.1062 (16)	−0.0069 (11)	−0.0110 (12)	0.0232 (12)
C5	0.0716 (11)	0.0487 (9)	0.0526 (9)	−0.0029 (8)	0.0101 (8)	0.0012 (7)
C6	0.0856 (13)	0.0656 (12)	0.0772 (12)	−0.0033 (10)	0.0315 (10)	0.0035 (9)
C7	0.0588 (10)	0.0628 (11)	0.0595 (9)	−0.0036 (8)	0.0162 (8)	0.0009 (8)
C8	0.0745 (12)	0.0604 (11)	0.0696 (11)	0.0016 (9)	0.0112 (9)	−0.0073 (8)
C9	0.0724 (12)	0.0521 (10)	0.0809 (12)	−0.0102 (9)	−0.0011 (9)	0.0066 (9)
C10	0.0684 (12)	0.0802 (13)	0.0709 (12)	−0.0198 (10)	0.0111 (9)	0.0164 (10)
C11	0.0672 (11)	0.0787 (12)	0.0552 (9)	−0.0115 (9)	0.0149 (8)	0.0008 (8)
C12	0.0512 (9)	0.0529 (9)	0.0517 (8)	−0.0048 (7)	0.0059 (7)	0.0057 (7)
C13	0.0522 (9)	0.0521 (9)	0.0507 (8)	−0.0033 (7)	0.0099 (7)	0.0008 (7)
C14	0.0576 (9)	0.0484 (9)	0.0559 (9)	−0.0010 (7)	0.0117 (7)	−0.0049 (7)
C15	0.0517 (9)	0.0481 (9)	0.0597 (9)	0.0047 (7)	0.0132 (7)	0.0000 (7)
C16	0.0429 (8)	0.0542 (9)	0.0544 (9)	0.0022 (7)	0.0126 (6)	−0.0010 (7)
C17	0.0558 (9)	0.0525 (10)	0.0606 (9)	−0.0009 (8)	0.0092 (7)	−0.0085 (7)
C18	0.0595 (10)	0.0469 (9)	0.0635 (10)	0.0054 (7)	0.0080 (8)	0.0017 (7)
C19	0.0437 (8)	0.0570 (10)	0.0589 (9)	−0.0002 (7)	0.0118 (7)	−0.0057 (7)
C20	0.0462 (9)	0.0565 (10)	0.0612 (9)	−0.0058 (7)	0.0098 (7)	−0.0016 (7)
C21	0.0413 (8)	0.0512 (9)	0.0588 (9)	−0.0006 (7)	0.0141 (7)	−0.0009 (7)
C22	0.0528 (9)	0.0501 (9)	0.0619 (9)	−0.0073 (7)	0.0139 (7)	−0.0040 (7)
C23	0.0491 (8)	0.0484 (9)	0.0592 (9)	−0.0017 (7)	0.0193 (7)	−0.0003 (7)
C24	0.0558 (9)	0.0552 (10)	0.0573 (9)	−0.0034 (7)	0.0135 (7)	−0.0001 (7)
C25	0.0471 (8)	0.0459 (8)	0.0597 (9)	−0.0019 (7)	0.0068 (7)	0.0021 (7)
C26	0.0476 (8)	0.0490 (9)	0.0646 (9)	0.0025 (7)	0.0089 (7)	−0.0003 (7)
C27	0.0488 (9)	0.0758 (12)	0.0828 (12)	−0.0002 (8)	0.0192 (8)	0.0162 (9)
C28	0.0809 (13)	0.0588 (11)	0.0841 (13)	−0.0143 (9)	−0.0039 (10)	−0.0047 (9)
C29	0.0636 (10)	0.0501 (9)	0.0652 (10)	−0.0030 (8)	0.0189 (8)	0.0023 (7)
C30	0.0869 (14)	0.0490 (10)	0.0813 (12)	−0.0072 (10)	0.0173 (10)	0.0056 (9)
C31	0.0738 (12)	0.0497 (10)	0.0730 (12)	−0.0035 (9)	0.0215 (9)	0.0079 (8)

### Geometric parameters (Å, °)

**Table:**

N1—C13	1.405 (2)	C15—H15	0.9300
N1—C5	1.418 (2)	C16—C17	1.395 (2)
N1—C12	1.431 (2)	C16—C19	1.453 (2)
N2—C30	1.141 (2)	C17—C18	1.378 (2)
N3—C31	1.143 (2)	C17—H17	0.9300
C1—C2	1.354 (4)	C18—H18	0.9300
C1—C6	1.392 (3)	C19—C20	1.342 (2)
C1—H1	0.9300	C19—H19	0.9300
C2—C3	1.347 (4)	C20—C21	1.435 (2)
C2—H2	0.9300	C20—H20	0.9300
C3—C4	1.375 (3)	C21—C22	1.358 (2)
C3—H3	0.9300	C21—C26	1.498 (2)
C4—C5	1.374 (3)	C22—C23	1.420 (2)
C4—H4	0.9300	C22—H22	0.9300
C5—C6	1.374 (2)	C23—C29	1.375 (2)
C6—H6	0.9300	C23—C24	1.494 (2)
C7—C8	1.373 (2)	C24—C25	1.533 (2)
C7—C12	1.375 (2)	C24—H24A	0.9700
C7—H7	0.9300	C24—H24B	0.9700
C8—C9	1.373 (3)	C25—C27	1.520 (2)
C8—H8	0.9300	C25—C28	1.524 (2)
C9—C10	1.365 (3)	C25—C26	1.531 (2)
C9—H9	0.9300	C26—H26A	0.9700
C10—C11	1.379 (2)	C26—H26B	0.9700
C10—H10	0.9300	C27—H27A	0.9600
C11—C12	1.381 (2)	C27—H27B	0.9600
C11—H11	0.9300	C27—H27C	0.9600
C13—C18	1.392 (2)	C28—H28A	0.9600
C13—C14	1.397 (2)	C28—H28B	0.9600
C14—C15	1.366 (2)	C28—H28C	0.9600
C14—H14	0.9300	C29—C31	1.428 (3)
C15—C16	1.402 (2)	C29—C30	1.428 (3)
			
C13—N1—C5	122.75 (13)	C17—C18—C13	120.40 (15)
C13—N1—C12	118.53 (12)	C17—C18—H18	119.8
C5—N1—C12	118.29 (13)	C13—C18—H18	119.8
C2—C1—C6	121.1 (2)	C20—C19—C16	125.98 (15)
C2—C1—H1	119.4	C20—C19—H19	117.0
C6—C1—H1	119.4	C16—C19—H19	117.0
C3—C2—C1	119.4 (2)	C19—C20—C21	126.94 (15)
C3—C2—H2	120.3	C19—C20—H20	116.5
C1—C2—H2	120.3	C21—C20—H20	116.5
C2—C3—C4	120.8 (2)	C22—C21—C20	119.28 (14)
C2—C3—H3	119.6	C22—C21—C26	119.98 (14)
C4—C3—H3	119.6	C20—C21—C26	120.72 (14)
C5—C4—C3	120.8 (2)	C21—C22—C23	123.27 (14)
C5—C4—H4	119.6	C21—C22—H22	118.4
C3—C4—H4	119.6	C23—C22—H22	118.4
C4—C5—C6	118.46 (17)	C29—C23—C22	121.22 (14)
C4—C5—N1	119.87 (17)	C29—C23—C24	120.23 (14)
C6—C5—N1	121.62 (16)	C22—C23—C24	118.50 (13)
C5—C6—C1	119.5 (2)	C23—C24—C25	114.28 (13)
C5—C6—H6	120.3	C23—C24—H24A	108.7
C1—C6—H6	120.3	C25—C24—H24A	108.7
C8—C7—C12	120.24 (16)	C23—C24—H24B	108.7
C8—C7—H7	119.9	C25—C24—H24B	108.7
C12—C7—H7	119.9	H24A—C24—H24B	107.6
C9—C8—C7	120.29 (17)	C27—C25—C28	109.77 (14)
C9—C8—H8	119.9	C27—C25—C26	110.51 (14)
C7—C8—H8	119.9	C28—C25—C26	109.03 (13)
C10—C9—C8	119.75 (17)	C27—C25—C24	110.18 (13)
C10—C9—H9	120.1	C28—C25—C24	108.51 (13)
C8—C9—H9	120.1	C26—C25—C24	108.80 (13)
C9—C10—C11	120.45 (17)	C21—C26—C25	113.60 (12)
C9—C10—H10	119.8	C21—C26—H26A	108.8
C11—C10—H10	119.8	C25—C26—H26A	108.8
C10—C11—C12	119.83 (17)	C21—C26—H26B	108.8
C10—C11—H11	120.1	C25—C26—H26B	108.8
C12—C11—H11	120.1	H26A—C26—H26B	107.7
C7—C12—C11	119.43 (15)	C25—C27—H27A	109.5
C7—C12—N1	120.57 (14)	C25—C27—H27B	109.5
C11—C12—N1	119.96 (14)	H27A—C27—H27B	109.5
C18—C13—C14	118.16 (14)	C25—C27—H27C	109.5
C18—C13—N1	121.87 (14)	H27A—C27—H27C	109.5
C14—C13—N1	119.96 (14)	H27B—C27—H27C	109.5
C15—C14—C13	120.86 (14)	C25—C28—H28A	109.5
C15—C14—H14	119.6	C25—C28—H28B	109.5
C13—C14—H14	119.6	H28A—C28—H28B	109.5
C14—C15—C16	121.88 (14)	C25—C28—H28C	109.5
C14—C15—H15	119.1	H28A—C28—H28C	109.5
C16—C15—H15	119.1	H28B—C28—H28C	109.5
C17—C16—C15	116.57 (14)	C23—C29—C31	122.11 (15)
C17—C16—C19	120.59 (14)	C23—C29—C30	121.35 (15)
C15—C16—C19	122.84 (14)	C31—C29—C30	116.48 (15)
C18—C17—C16	122.08 (15)	N2—C30—C29	178.8 (2)
C18—C17—H17	119.0	N3—C31—C29	179.1 (2)
C16—C17—H17	119.0		
			
C6—C1—C2—C3	−0.5 (4)	C14—C15—C16—C17	1.5 (2)
C1—C2—C3—C4	−0.1 (4)	C14—C15—C16—C19	−177.95 (14)
C2—C3—C4—C5	0.2 (4)	C15—C16—C17—C18	−2.3 (2)
C3—C4—C5—C6	0.2 (3)	C19—C16—C17—C18	177.10 (14)
C3—C4—C5—N1	177.9 (2)	C16—C17—C18—C13	0.9 (2)
C13—N1—C5—C4	144.71 (18)	C14—C13—C18—C17	1.4 (2)
C12—N1—C5—C4	−42.9 (2)	N1—C13—C18—C17	−177.36 (14)
C13—N1—C5—C6	−37.7 (2)	C17—C16—C19—C20	−169.57 (15)
C12—N1—C5—C6	134.71 (17)	C15—C16—C19—C20	9.8 (2)
C4—C5—C6—C1	−0.7 (3)	C16—C19—C20—C21	179.76 (14)
N1—C5—C6—C1	−178.40 (17)	C19—C20—C21—C22	−172.13 (15)
C2—C1—C6—C5	0.9 (3)	C19—C20—C21—C26	6.3 (2)
C12—C7—C8—C9	0.0 (3)	C20—C21—C22—C23	177.62 (13)
C7—C8—C9—C10	0.1 (3)	C26—C21—C22—C23	−0.8 (2)
C8—C9—C10—C11	−0.7 (3)	C21—C22—C23—C29	−175.45 (14)
C9—C10—C11—C12	1.3 (3)	C21—C22—C23—C24	2.0 (2)
C8—C7—C12—C11	0.5 (3)	C29—C23—C24—C25	−158.00 (14)
C8—C7—C12—N1	−177.28 (15)	C22—C23—C24—C25	24.5 (2)
C10—C11—C12—C7	−1.2 (3)	C23—C24—C25—C27	72.25 (18)
C10—C11—C12—N1	176.63 (15)	C23—C24—C25—C28	−167.55 (13)
C13—N1—C12—C7	116.95 (17)	C23—C24—C25—C26	−49.05 (18)
C5—N1—C12—C7	−55.8 (2)	C22—C21—C26—C25	−26.7 (2)
C13—N1—C12—C11	−60.8 (2)	C20—C21—C26—C25	154.85 (14)
C5—N1—C12—C11	126.46 (17)	C27—C25—C26—C21	−71.15 (17)
C5—N1—C13—C18	−33.5 (2)	C28—C25—C26—C21	168.12 (13)
C12—N1—C13—C18	154.15 (15)	C24—C25—C26—C21	49.95 (18)
C5—N1—C13—C14	147.73 (15)	C22—C23—C29—C31	178.69 (14)
C12—N1—C13—C14	−24.6 (2)	C24—C23—C29—C31	1.2 (2)
C18—C13—C14—C15	−2.3 (2)	C22—C23—C29—C30	1.7 (2)
N1—C13—C14—C15	176.54 (14)	C24—C23—C29—C30	−175.73 (15)
C13—C14—C15—C16	0.8 (2)		
